# Waist-to-height ratio is a useful index for nonalcoholic fatty liver disease in children and adolescents: a secondary data analysis

**DOI:** 10.1186/s12889-017-4868-5

**Published:** 2017-10-30

**Authors:** Ming-Shyan Lin, Tsai-Hui Lin, Su-Er Guo, Ming-Horng Tsai, Ming-Shin Chiang, Tung-Jung Huang, Mei-Yen Chen

**Affiliations:** 1 Department of Cardiology, Chang Gung Memorial Hospital, Yunlin, Taiwan; 2 Department of Internal Medicine and Traditional Chinese Medicine, China Medical University Hospital, Taichung, Taiwan; 3 Graduate Institute of Nursing, Chang Gung University of Science and Technology, Chiayi, Taiwan; 4 Chronic Diseases & Health Promotion Research Center, Research Center for Industry of Human Ecology, Chang Gung University of Science and Technology, Taoyuan, Taiwan; 5 Department of Pediatrics, Chang Gung Memorial Hospital, Yunlin, Taiwan; 6 Department of Hepato-Gastroenterology, Chang Gung Memorial Hospital, Yunlin, Taiwan; 7 Department of Pulmonary Disease, Chang Gung Memorial Hospital, Yunlin, Taiwan; 8 Department of Respiratory Care, Chang Gung University of Science and Technology, Chiayi, Taiwan; 9 College of Nursing, Chang Gung University of Science and Technology, Chiayi, Taiwan; 10 Department of Nursing, Chang Gung University, Taoyuan, Taiwan; 11 Research Fellow, Chang Gung Memorial Hospital, Chiayi, Taiwan

**Keywords:** Childhood obesity, cutoff value, nonalcoholic fatty liver disease, waist-to-height ratio, waist-to-hip ratio

## Abstract

**Background:**

Nonalcoholic fatty liver disease (NAFLD) is a global problem and pediatric obesity has risen dramatically. Early NAFLD might progress to nonalcoholic steatohepatitis (NASH) or liver cirrhosis and significantly increase liver disease-related mortality. We looked for NAFLD predictors in children and adolescents.

**Methods:**

This community-based, cross-sectional study ran from December 2012 to September 2013 in southwestern Taiwan. Children <10 and >19 years old, with detected hepatic diseases, or who drank alcohol were excluded. The diagnosis of NAFLD was based on ultrasound: age, sex, anthropometric measurements, and laboratory data were evaluated for associated risks by using logistic regression analysis. Receiver operating characteristic (ROC) curves were used to determine cutoff values.

**Results:**

We enrolled one thousand, two hundred and ten children (594 males; 616 females; mean age: 15.5 ± 2.8 years). Age, anthropometric measurements, and laboratory data were significantly higher in children with NAFLD. The association between NAFLD and the waist-to-height ratio (WHtR) was significant (adjusted odds ratio: 2.6; 95% confidence interval: 1.909-3.549; *P* < 0.001). It indicated highly suspicion of NAFLD (sensitivity: 70.1%; specificity 76.9%) when the WHtR for children and adolescents is above the cutoff value of 0.469.

**Conclusions:**

The WHtR might be a powerful index of the severity of pediatric NAFLD.

## Background

Non-alcoholic fatty liver disease (NAFLD) is an emerging health problem associated with childhood obesity and adult metabolic syndrome (MetS) [1]. NAFLD might be an early manifestation of insulin resistance [2], and body fat distribution is important because high abdominal adiposity reflects excess central and visceral adipose tissue (VAT), which is intimately associated with metabolic disease and adverse outcomes in adulthood [3]. Recent review study [4] claimed that the mean prevalence of NAFLD in general pediatric population was 7.6% lower than the rate (10.2% in Asian subjects) according to prior autopsy study [5]. Another Taiwan investigation [6] also reported higher incidence of pediatric NAFLD than the result from systemic review (76% vs. 34.2% in obese group). Clinical and pathological presentations of NAFLD are various, such as those in asymptomatic hepatitis, fibrosis, or cirrhosis patients, because of a lack of a definitive diagnosis and screening guidelines. Therefore, simple, accurate, reproducible, and inexpensive screening tools for early NAFLD are essential for children and adolescents.

Liver biopsy is the gold standard diagnostic tool for NAFLD, but it is expensive, invasive, risky, and infeasible in an annual health check-up. Ultrasound is a popular screening tool for patients with an abnormal liver function test, which varies without consistent values or upper normal thresholds in children. Body Mass Index (BMI) and waist circumference are anthropometric measurements widely used to evaluate the effects of obesity on metabovascular risk factors and NAFLD [7]. Contrary to waist circumference and BMI, the values of waist-to-hip ratio (WHR) and waist-to-height ratio (WHtR) have the advantages without requiring population-specific reference tables or changes in body composition with growth and development. Therefore, the WHR appears to be more strongly associated with abdominal obesity than does BMI [8], and the WHR is strongly correlated with VAT [9]. Moreover, one meta-analysis [10] showed evidence to support the superiority of measuring centralized obesity, especially the WHtR, rather than BMI and waist circumference, for detecting cardiovascular risk factors both in men and in women. The WHtR has been proposed as a more reliable anthropometric index for detecting childhood obesity [11], body fat percentage [12], and Lee et al. [13] also reported the effects of the WHtR on visceral fat and metabolic components in Korean children and adolescents. However, no studies have evaluated the association between the WHtR and NAFLD in Asian pediatric populations. Therefore, we try to assess the reliability of anthropometric indices for hepatosteatosis in Taiwanese children and adolescents.

## Methods

### Design

This study used a correlational cross-sectional design in which anthropometric measure and NAFLD were assessed concurrently in prepubertal children and adolescents. Ethical approval was provided by the institutional review board ethical committee (Chang-Gung Memorial Hospital Ethics Committee No 102-4399B).

### Sample

**Fig. 1:**
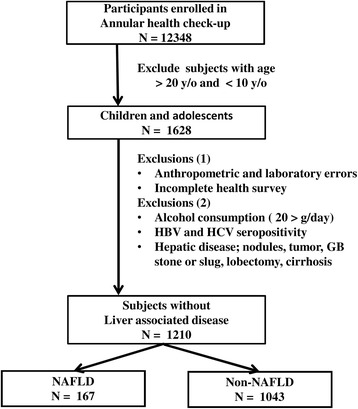
Flow chart of participant selection. GB, gallbladder; HCV, hepatitis C virus; HBV, hepatitis B virus; NAFLD, nonalcoholic fatty liver disease

From December 2012 to September 2013 in Yunlin County, Taiwan, 12,348 residents underwent a community-based annual health checkup. We selected 10- to 19-year-olds for the study and obtained the informed consent from all of them. The exclusion criteria were: (1) < 10 or >19 years old; (2) incomplete health survey, anthropometric measurements, or laboratory data; (3) hepatitis B (HBV), hepatitis C (HCV), or both; (4) prior hepatic surgery; (5) habitual alcohol drinking; (6) liver cirrhosis, nodules, or tumors revealed by ultrasound scan. We finally enrolled 1210 participants (Fig. 1).

### Anthropometric assessment

A noninvasive oscillometric monitor (Omega 1400; Invivo Research Inc., Orlando, FL, USA) was calibrated and then was used to measure blood pressure (BP) with a standard procedure and appropriate cuff size. Every participant’s BP was measured twice and recorded. If the difference between first and second BP value was >10 mmHg, BP was measured a third time. The mean BP value was calculated in twice closest BP value and hypertension was defined as an average systolic or diastolic blood pressure level that was in the 95th percentile or greater based on at least three separate readings under guideline of the National High Blood Pressure Education Program (NHBPEP) [14].

Height (Ht), weight (Wt), and body mass index (BMI) were anthropometric measurements and plotted on Taiwan growth curves [15]. Height and body weight was measured under light clothes and bare feet. Body Mass Index (BMI) was calculated for each participantby using the standard formula (weight in kilograms divided by square of the height in meters). Based on the nationwide standard set by the Taiwan Bureau of Health Promotion (BHP, 2013), BMI was plotted on the age and sex-specific cutoff points to define the body size of children.

An anthropometric tape was used to measure waist circumference (WC) and hip circumference. WC was measured at the level midway between the lowest rib margin and the iliac crest. Hip circumference was measured at the maximum protuberance of the buttocks in a standing position.

### Laboratory assays

Venous blood samples were drawn from all participants after a 12-h overnight fast and delivered to the laboratory on the same day. The serum collected in the morning was measured (7600 Chemistry Analyzer; Hitachi Medical Corp., Tokyo, Japan) to detect serum uric acid, triglyceride (TG), total cholesterol (TC), high-density lipoprotein cholesterol (HDL-C), low-density lipoprotein cholesterol (LDL-C), fasting plasma glucose (FPG), alanine aminotransferase (ALT), aspartate aminotransferase (AST), gamma-glutamyl-transferase (GGT)), and creatinine (Cr). Seropositivity of antibodies to HCV was assessed by means of an electrochemiluminescence immunoassay and seropositivity to HBV was assessed with a semiquantitative determination of hepatitis B surface antigens by using a sandwich radioimmunoassay (Elecsys E-170; Cobas Analyzer; Roche Diagnostics, Indianapolis, IN).

### Lifestyle and medical history

Alcohol drinking, betel nut chewing, and cigarette smoking habits were evaluated. Participants were classified as *non-user* (never drank alcohol/chewed betel-nut/smoked cigarettes, or had not drunk/chewed/smoked for the previous year), or *current user* (currently drinking/chewing/smoking).

### Abdominal ultrasonography

Right upper quadrant ultrasound was performed on one of three units: an Aloka SSD 4000 (Hitachi Aloka Medical Ltd., Tokyo, Japan) with a UST-979-3.5 curved array transducer; CGM OPUS 5000 (Chang Gung Medical Technology Co., Ltd., Taipei, Taiwan) with a CLA35 curved array transducer; or an Acuson S2000 Ultrasound System (Siemens, Malvern, PA, USA) by using a C4-1 MHz curved array transducer. Technical parameters were adjusted for each patient with the standard protocol for a right upper quadrant ultrasound examination. The liver was considered normal if the echo-texture was homogeneous without acoustic attenuation, the portal veins were visible, the diaphragm was well visualized, and echogenicity was similar or slightly higher than that of the renal parenchyma. The diagnosis of fatty liver was based on the differences between the echogenicity in the liver and the kidney, vascular blurring of the hepatic vein trunk, and deep attenuation in the right hepatic lobe. The severity of fatty liver change was classified according to the standardized ultrasonographic criteria: grade 0, normal liver, a normal echo texture and absence of fatty change; grade 1, mild fatty liver change, a mildly increased echogenicity in the parenchyma comparing with the kidney, and slightly impaired visualization of intrahepatic vessels and diaphragm; grade 2, medium grade diffuse increased in hepatic echogenicity, mild deterioration in the image of the diaphragm and intrahepatic vessels; grade 3, moderate-to-severe fatty liver change, marked increase in fine echoes in the parenchyma with poor or non-visualization of the intrahepatic vessel borders, diaphragm, and posterior right lobe of the liver. The criteria for severe fatty liver changes were obvious and unambiguous. All images were reviewed on a picture archiving and communication system (Centricity PACS; GE Healthcare, Allendale, NJ, USA).

### Definitions

(1) NAFLD was defined as (a) ultrasonographic evidence of hepatic steatosis, and (b) absent current alcohol consumption, hereditary disorders, or using steatogenic medication.

(2) Obesity was defined when BMI was ≥ the 95th percentile [15].

(3) Components of International Diabetes Foundation defined MetS [16]: The criterion of waist circumference for MetS was infeasible because of absent update references in Taiwan. For children 10-16 years old, MetS components included triglyceride ≥150 (mg/dL), HDL < 40 (mg/dL), SBP ≥ 130 mmHg or DBP ≥ 85 mmHg and fasting glucose ≥100 mg/dL. For children >16 years old, MetS criteria were triglyceride ≥150 (mg/dL), HDL < 40 mg/dL in males and <50 mg/dL in females, SBP ≥ 130 mmHg or DBP ≥ 85 mmHg and fasting glucose ≥100 mg/dL.

### Statistical analysis

SPSS 19.0 (SPSS, Inc., Chicago, IL, USA) was used for all analyses. Numeric variables were expressed as mean ± standard deviation (SD) and categorical variables as number (percent). Continuous data for the study group and comparison group were compared using Student’s *t* test. Categorical data were analyzed using χ^2^ or Fisher’s Exact tests, and 95% CIs and multivariate logistic regression analyses were used to identify the best subset of independent predictors. All tests were two-sided and significance was set at *P* < 0.05. A multiple logistic regression analysis was used to identify the variables affecting the dependent variable NAFLD (using odds ratios [ORs] and 95% CIs). The independent variables included in the analyses were age groups, sex, and BMI categories.

The cutoff value for the risk score was determined by using the receiver operating characteristic (ROC) curve procedure. The ROC curve was plotted for NAFLD; sensitivity was plotted on the *y*-axis and the false-positive rate (1−specificity) was plotted on the *x*-axis.

## Results

### Participant demographics

The data of 616 (50.1%) females and 594 (49.1%) males (mean (SD) age: 15.5 (2.8) years) were analyzed (Table 1). The overall prevalence of obesity in the study population was 16.3%. Those obese subjects were predominant in male gender (male vs female 62.4% vs. 37.6%; *P* < 0.001) with similar portion of smoking and betel nut chewing compared with non-obese group. Higher BMI (28.3 ± 3.2 vs. 19.9 ± 2.7; *P* < 0.001), body weight (74.7 ± 15.5 vs. 51.7 ± 11.6; *P* < 0.001), and waist circumference (73.5 ± 12.6 vs. 71.52 ± 10.44; *P* = 0.020) were observed in obese group; however, the BP or laboratory data were insignificantly different.

**Table 1:** Baseline characteristics of the study participants by adiposity

Variable	Total	Obese	Non-obese	*P*
	(*N* = 1210)	(*n* = 197)	(*n* = 1013)	
Age (years)	15.5 ± 2.8	15.2 ± 3.0	15.6 ± 2.8	0.063
Male, n (%)	594 (49.1)	123 (62.4)	471 (46.5)	< 0.001
Anthropometric assessment				
Body weight (kg)	55.5 ± 14.9	74.7 ± 15.5	51.7 ± 11.6	< 0.001
BMI (kg/m^2^)	21.3 ± 4.2	28.3 ± 3.2	19.9 ± 2.7	< 0.001
Waist (cm)	71.8 ± 10.8	73.5 ± 12.6	71.5 ± 10.4	0.060
Waist-to-height ratio	0.8 ± 0.1	0.5 ± 0.1	0.4 ± 0.1	0.123
Waist-hip ratio	0.45 ± 0.06	0.81 ± 0.08	0.80 ± 0.07	0.236
Systolic BP (mmHg)	114.2 ± 18.5	115.8 ± 19.5	113.9 ± 18.3	0.208
Diastolic BP (mmHg)	67.9 ± 12.9	68.5 ± 13.9	67.9 ± 13.8	0.503
Pulse pressure (mmHg)	46.3 ± 14.2	47.2 ± 14.6	46.1 ± 14.1	0.304
Lifestyle history				
Smoking, n (%)	29 (2.4)	6 (3.0)	23 (2.3)	0.515
Betel nut chewing, n (%)	6 (0.5)	2 (0.9)	4 (0.4)	0.335
Biochemistry data				
Creatinine (mg/dL)	0.79 ± 0.17	0.77 ± 0.16	0.79 ± 0.17	0.177
Fasting blood glucose (mg/dL)	90.8 ± 8.6	91.1 ± 7.2	90.7 ± 8.8	0.608
ALT (mg/dL)	17.0 ± 16.4	18.1 ± 19.8	16.8 ± 19.7	0.336
AST (mg/dL)	19.1 ± 7.4	19.6 ± 9.6	19.0 ± 6.9	0.328
Uric acid (mg/dL)	5.73 ± 1.43	5.71 ± 1.51	5.73 ± 1.42	0.782
GGT (U/L)	15.2 ± 10.3	16.5 ± 14.3	14.9 ± 9.4	0.063
Lipid profiles				
LDL (mg/dL)	95.5 ± 25.7	95.6 ± 27.4	95.5 ± 25.3	0.957
VLDL (mg/dL)	15.6 ± 7.7	15.8 ± 7.5	15.5 ± 7.7	0.628
HDL (mg/dL)	57.9 ± 11.4	58.1 ± 11.9	57.9 ± 11.3	0.821
Cholesterol (mg/dL)	162.3 ± 27.3	162.8 ± 29.4	162.2 ± 26.9	0.777
TG (mg/dL)	78.2 ± 41.2	80.1 ± 44.8	77.9 ± 40.4	0.490
GGT (U/L)	15.2 ± 10.3	16.5 ± 14.3	14.9 ± 9.4	0.063
NAFLD, n (%)	167 (13.8)	35 (17.8)	132 (13.0)	0.078

*Abbreviation*: *ALT* aspartate aminotransferase, *AST* alanine aminotransferase, *NAFLD* non-alcoholic fatty liver disease, *BW* body weight, *BMI* basal metabolic index, *BP* blood pressure, *GGT* gamma-glutamyl aminotransferase, *LDL* low-density lipoprotein cholesterol, *VLDL* very-low-density lipoprotein cholesterol, *HDL* high-density lipoprotein cholesterol, *TG* triglycerides

### Factors associated with ultrasonically diagnosed NAFLD

**Fig. 2:**
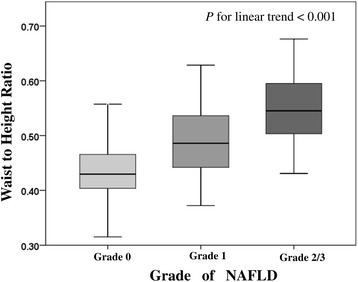
Box plots showing comparisons between waist to height ratio (WHtR) and grade of NAFLD. In each group, the central line indicates the median value, the upper and lower lines represent the upper and lower quartiles, and the crosses show the minimum and maximum values. The WHtR differs significantly with the grade of NAFLD (Grade 0, 0.70; Grade 1, 0.97; Grade 2/3, 1.46; linear trend *P* < 0.001).NAFLD, non-alcoholic fatty liver disease

**Fig. 3:**
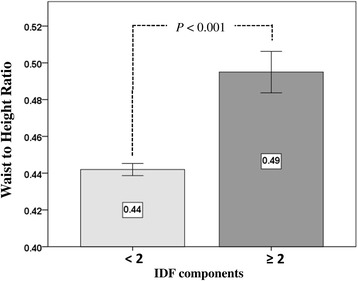
The differences between mean values of WHtR and components of IDF defined MetS. IDF, International Diabetes Foundation; WHtR, waist-to-height ratio

The participants were divided into two groups based on whether they had been ultrasonically proved fatty liver, and, according to ultrasonographic criteria, 167 (13.8%) of the participants were diagnosed with NAFLD (Table 2). The percentage differences between NAFLD participants was not significant based on sex (male: 54.5% vs. female: 48.2%; OR: 1.285; 95% CI: 0.926-1.784), age (18.0 ± 1.9 vs. 15.1 ± 2.8; OR: 1.823; 95% CI: 1.627-2.042), or obesity (21.0% vs. 15.5%; OR: 1.442; 95% CI: 0.958-2.170) in comparison with the non-NAFLD group. Additionally, NAFLD group members had a significantly greater WHR (0.86 ± 0.08 vs. 0.79 ± 0.07; OR: 2.556; 95% CI: 2.106-3.104), greater WHtR (0.51 ± 0.07 vs. 0.44 ± 0.05; OR: 3.885; 95% CI: 2.331-5.233) as well as higher BMI than those in the non-NAFLD group. Although serum Cr and fasting blood glucose levels were non-significant between groups, NAFLD group members had significantly higher BPs and lipid profiles (LDL, VLDL, TG, and TC levels); ALT, AST, uric acid, and GGT levels; and lower HDL levels than non-NAFLD group members. Participants with higher grade NAFLD also had a significantly larger WHtR. (Figure 2) In addition, participants with more than two IDF-defined MetS components had a significantly larger WHtR than subjects with less than two points (0.49 vs 0.44; *P* < 0.001) (Fig. 3).

**Table 2:** Baseline characteristics of the study participants by NAFLD

Variable	NAFLD (*n* = 167)	Non-NAFLD (*n* = 1043)	Crude OR (95% CI)	*P*
Age (years)	18.0 ± 1.9	15.1 ± 2.8	1.823 (1.627-2.042)	< 0.001
Male, n %	91 (54.5)	503 (48.2)	1.285 (0.926-1.784)	0.133
Anthropometric Assessment				
BMI (kg/m^2^)	23.0 ± 4.7	21.0 ± 4.0	1.109 (1.070-1.150)	< 0.001
Waist-hip ratio	0.86 ± 0.08	0.79 ± 0.07	2.556 (2.106-3.104)	< 0.001
Waist-to-height ratio	0.51 ± 0.07	0.44 ± 0.05	3.885 (2.331-5.233)	< 0.001
Systolic BP (mmHg)	121.2 ± 19.4	113.1 ± 18.1	1.023 (1.014-1.031)	< 0.001
Diastolic BP (mmHg)	71.8 ± 14.3	67.4 ± 12.7	1.024 (1.012-1.036)	< 0.001
Pulse pressure (mmHg)	49.4 ± 14.5	45.8 ± 14.1	1.017 (1.006-1.028)	< 0.001
Biochemistry data				
Creatinine (mg/dL)	0.76 ± 0.17	0.79 ± 0.17	0.364 (0.132-0.999)	0.050
ALT (mg/dL)	30.0 ± 27.5	14.9 ± 12.7	1.048 (1.037-1.060)	< 0.001
AST (mg/dL)	22.2 ± 10.3	18.7 ± 6.7	1.048 (1.028-1.069)	< 0.001
Uric acid (mg/dL)	6.35 ± 1.59	5.63 ± 1.38	1.403 (1.254-1.570)	< 0.001
GGT (U/L)	22.8 ± 17.4	18.7 ± 6.7	1.068 (1.050-1.085)	< 0.001
Lipid profiles				
LDL-C (mg/dL)	104.4 ± 30.4	94.1 ± 24.5	1.014 (1.008-1.020)	< 0.001
VLDL (mg/dL)	19.4 ± 10.6	14.9 ± 6.9	1.060 (1.041-1.080)	< 0.001
HDL-C mg/dL	54.4 ± 11.5	58.5 ± 11.3	0.966 (0.951-0.982)	< 0.001
Cholesterol-C mg/dL	168.6 ± 31.4	161.3 ± 26.5	1.009 (1.004-1.015)	0.001
Triglyceride (mg/dL)	98.8 ± 59.9	74.9 ± 36.2	1.011 (1.007-1.014)	< 0.001

*Abbreviation*: *ALT* aspartate aminotransferase, *AST* alanine aminotransferase, *NAFLD* non-alcoholic fatty liver disease, *BW* body weight, *BMI* basal metabolic index, *BP* blood pressure, *GGT* gamma-glutamyl aminotransferase, *LDL* low-density lipoprotein cholesterol, *VLDL* very-low-density lipoprotein cholesterol, *HDL* high-density lipoprotein cholesterol, *TG* triglycerides

Hierarchical multiple logistic regression showed that the WHtR was most significantly associated with NAFLD (adjusted OR: 2.600; 95% CI: 1.909-3.549; *P* < 0.001) after final adjustments (Table 3).

**Table 3:** Association of the WHtR and WHR with NAFLD in four adjusted multiple logistic regression analysis models

	Explanatory variables of primary interest (per SD increase)					
	Waist-Hip Ratio			Waist-to-Height Ratio		
Model	OR	95% CI	*P*	OR	95% CI	*P*
Model 1	1.857	1.504-2.294	< 0.001	2.725	2.090-3.551	< 0.001
Model 2	1.800	1.456-2.225	< 0.001	2.718	2.064-3.581	< 0.001
Model 3	1.563	1.262-1.936	< 0.001	2.610	1.921-3.547	< 0.001
Model 4	1.562	1.259-1.936	< 0.001	2.600	1.909-3.549	< 0.001

*SD* standard deviation, *OR* odds ratio, *CI* confidence interval

Model 1: adjusted for age and sex

Model 2: further adjusted for body mass index and pulse pressure

Model 3: further adjusted for serum creatinine, uric acid, GGT, ALT, AST

Model 4: further adjusted for lipid profiles

### The area under the ROC curve (AUC) of the WHtR and the WHR for NAFLD

**Fig. 4:**
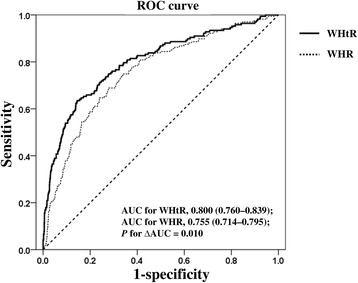
ROC curve analysis to determine the cutoff value of the WHtR and WHR for predicting NAFLD. The cutoff value of the WHtR for predicting NAFLD is 0.469 (sensitivity: 70.1%; specificity: 76.9%). The area under the ROC curve (AUC) of the WHtR is 0.80 and of the WHR is 0.755; *P* = 0.010). NAFLD, nonalcoholic fatty liver disease; ROC, receiver operating characteristic; WHtR, waist-to-height ratio; WHR, waist-to-hip ratio

The WHtR AUC (0.80; CI: 0.760-0.839) predicting NAFLD was significantly higher than the WHR AUC (0.755; CI: 0.714-0.795) (Fig. 4). The cutoff value of the WHtR for children and adolescents was 0.469, which indicates a high risk of having NAFLD if it was above the value (sensitivity: 70.1%; specificity 76.9%).

## Discussion

Although many studies have evaluated the correlation between the WHtR and central obesity, this is the first study to reveal a strong relationship between the WHtR and NAFLD in children and adolescents. The participants with a higher WHtR value had a 2.6-fold higher risk of NAFLD (95% CI: 1.909-3.549; *P* < 0.001) per standard deviation (SD), and the cutoff value was 0.469 for 10- to 19-year-olds.

The prevalence of NAFLD in our participants was 13.8% in the total population, which was similar to the findings (10.2%) in the autopsy study [5]. The prevalence rate of NAFLD was higher in obese group; however, the rate was relatively lower than those reports of prior investigations [4, 6]. Differences might be associated with geographical region, diet habit and study sample size. In fact, Fishbein and colleagues [17] supported the notion that visceral adiposity was better than BW or BMI at predicting fatty liver. We also found nonsignificant metabovascular features in the obese group, but all these features of MetS were significant in participants with NAFLD. These findings were compatible with the Bogalusa Heart Study [18], which showed that the distribution of central fat in 5- to 17-year-olds, determined by using WC, and was associated with abnormal levels of TG, LDL-C, HDL-C, and insulin. Boyraz et al. [19] further reported that obese children with NAFLD had frequent MetS features. Schwimmer and colleagues [20] had previously shown that children with MetS had 5 times the odds of having NAFLD than those overweight and obese children without MetS. Additionally, pediatric MetS definition was limited because (1) all anthropometric assessments (BW, BMI, and WC) varied with age, gender, and ethnicity; and (2) MetS criteria could not be adequately defined because WC reference data were lacking update in many countries.

WHtR is a better predictor for NAFLD than the WHR in some reasons: (1) more accurate sagittal and upper abdominal fat distribution; (2) hip circumference reflects different body components, such as fat, muscle, as well as bone mass. A WHtR threshold level of 0.5 has been proposed and recently validated [21]. Weili and colleagues [12] suggested 0.445 as the WHtR cutoff value for overweight and 0.485 for obesity in Chinese children and adolescents. A study [22] in Brazil showed a WHtR cutoff value of from 0.42-0.45 for dyslipidemia stratified by sex. Lee et al. [13] reported WHtR cutoff range of 0.54-0.61 is corresponded to the visceral fat area (VFA) of Korean 10- to 15-year-olds and WHtR cutoff range of 0.51-0.56 is corresponded to the VFA of 16- to 18-year-olds. Tuan and colleagues [23] used dual-energy X-ray absorptiometry to report 5355 children at the 75th percentile had a WHtR between 0.5-0.537, which was strongly correlated with adiposity. However, the WHtR cutoff value for ultrasonically defined NAFLD has not been validated. We found that the WHtR cutoff value of 0.469 is corresponded to NAFLD in Taiwanese 10- to 19-year-olds. The cutoff value is similar to the prior validation studies but refills the gap for echogenic assessment in general health checkup.

Feldstein and colleagues [24] reported that the survival of children with NAFLD was significantly shorter than that of their peers without NAFLD. Thus predicting NAFLD was very essential for early intervention on childhood obesity and metabovascular complications. The association of insulin resistance syndrome and cardiovascular risk is not only related to the degree of obesity, but also appears to be critically dependent on body fat distribution [11, 25]. Ochiai and colleagues [26] had observed strong correlation of WHtR than BMI or WC with ALT levels, and higher value of WHtR was significantly correlated with more cardiometabolic risk factors [27], hypertension [28], and future mortality [29]. Additional large cohort studies are required to confirm that WHtR is associated with NAFLD and with subsequent adult metabovascular insults.

### Limitations

A liver biopsy has been a gold standard in the clinical diagnosis of NAFLD, but it is not well suited for screening or monitoring children because it is invasive and expensive, and it can cause complications in a pediatric physical examination. Ultrasonic sensitivity is acceptable, and interoperative differences can be reduced by experienced ultrasound users or by a review. A small regional study will limit the accuracy of the findings, but a large nationwide study will increase it. Nambiar and colleagues [30] had reported that error associated with the WHtR was “clinically and biologically acceptable”.

## Conclusion

Despite some limitations, our study showed that the WHtR is strongly associated with fatty liver in children and adolescents. In addition to dietary education and exercise to prevent childhood obesity, using the WHtR simply as a primary screening tool should increase early detection of NAFLD and assess cardiometabolic risks before puberty.

## Abbreviations

ALT: Alanine aminotransferase
AST: Aspartate aminotransferase
AUC: Area under the curve
BMI: Body mass index
FPG: Fasting plasma glucose
GGT: Gamma-glutamyl-transferase
HBV: Hepatitis B virus
HCV: Hepatitis C virus
HDL-C: High-density lipoprotein cholesterol
LDL-C: Low-density lipoprotein cholesterol
MetS: Metabolic syndrome
NAFLD: Non-alcoholic fatty liver disease
NASH: Nonalcoholic steatohepatitis
ROC: Receiver operating characteristic
TC: Total cholesterol
TG: Triglyceride
VAT: Visceral adipose tissue
VLDL: Very-low-density lipoprotein cholesterol
WHR: Waist to hip ratio
WHtR: Waist-to-height ratio
